# Potassium-Doped Amorphous Calcium Phosphate Nanoparticles Functionalized with Urea as a Sustainable Controlled-Release NPK Fertilizer

**DOI:** 10.1021/acsanm.6c02420

**Published:** 2026-07-23

**Authors:** Salem Ghribi, Lorenzo Degli Esposti, Blaire Steven, Nubia Zuverza-Mena, Jing Yuan, Jason C. White, Deb P. Jaisi, Alessio Adamiano, Michele Iafisco

**Affiliations:** † Institute of Science, Technology and Sustainability for Ceramics (ISSMC), National Research Council (CNR), Faenza, Ravenna 48018, Italy; ‡ Department of Mathematical, Physical and Computer Sciences, University of Parma, Parma 43124, Italy; § Dipartimento di Chimica E Chimica Industriale, 9302 Università Degli Studi di Genova, Genova 16146, Italy; ∥ 5758 Connecticut Agricultural Experiment Station, New Haven, Connecticut 06511, United States; ⊥ Interdisciplinary Science and Engineering Laboratory, Department of Plant and Soil Sciences, 5972 University of Delaware, Newark, Delaware 19716, United States; # Institute of Nanostructured Materials (ISMN), 9327 National Research Council (CNR), Bologna 40129, Italy

**Keywords:** amorphous calcium phosphate, controlled-release fertilizer, potassium doping, nutrient leaching reduction, sustainable agriculture

## Abstract

Conventional fertilizers are associated with substantial nutrient losses and environmental impacts, highlighting the need for more sustainable fertilization strategies. Here, we report the synthesis of potassium-doped amorphous calcium phosphate nanoparticles functionalized with urea (KACP-U) as a controlled-release nitrogen–phosphorus–potassium (NPK) nanofertilizer. Structural and compositional analyses confirmed the amorphous nature of the calcium phosphate matrix, effective potassium incorporation, and successful urea loading. Soil column leaching experiments under severe simulated rainfall conditions demonstrated that KACP-U significantly mitigates nutrient losses, with sustained urea release and a marked reduction of phosphorus, calcium, and potassium leaching by more than 1 order of magnitude compared with conventional fertilizers. Greenhouse experiments with corn (*Zea mays*) provided evidence that the controlled-release behavior of KACP-U observed in the leaching experiments was maintained under greenhouse conditions. KACP-U reduced phosphorus and calcium leaching by approximately 35% and urea (an N-containing compound) leaching by approximately 75%, with respect to a physical mixture of monocalcium phosphate, potassium chloride, and urea. At the same time, shoot biomass remained comparable to the control, while root biomass significantly increased by more than 47%. Notably, soil pH remained close to that of untreated soil, indicating a potentially lower risk of soil acidification compared with conventional nitrogen fertilizer. Results also indicate increased Ca and Mg accumulation in roots, by approximately 35 and 90%, respectively, compared with conventional fertilizer. This study underscores the potential of amorphous calcium phosphate–based nanofertilizers as a sustainable alternative to conventional NPK formulations.

## Graphical abstract

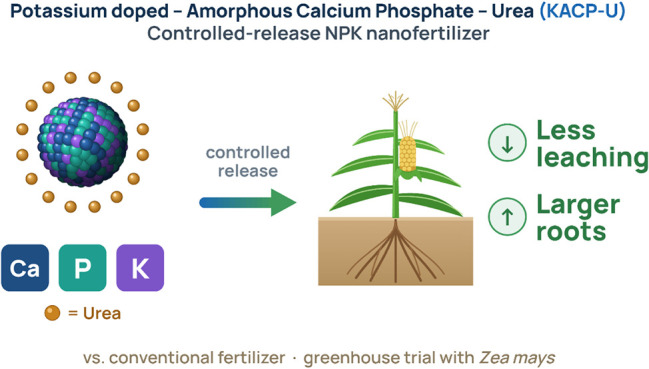

## Introduction

The challenge of increasing food production to meet global demand under the constraints imposed by the climate crisis has intensified the search for innovative strategies to enhance agricultural efficiency while adhering to sustainability principles. In line with the United Nations 2030 Agenda for Sustainable Development, improving crop yields while minimizing the environmental footprint of agricultural practices represents an urgent priority.^1^ One of the major obstacles to achieving this goal is the intrinsically low efficiency of conventional fertilizers, particularly those supplying nitrogen and phosphorus.^2–4^ Plants typically absorb less than 50% of applied nitrogen and less than 30% of phosphorus, with the remaining fractions lost through leaching and runoff, resulting in economic inefficiencies and significant ecological impacts.^5–7^

Nutrient leaching contributes to groundwater contamination and eutrophication, ultimately leading to the degradation of aquatic ecosystems.^4,8,9^ In addition, nitrogen-based fertilizers are associated with soil acidification and emission of greenhouse gases, including nitrous oxide.^5,10,11^ These issues underscore the need for advanced fertilizer formulations capable of enhancing nutrient retention, reducing losses, and synchronizing nutrient availability with plant uptake. In this context, nanotechnology has emerged as a promising approach to improve nutrient use efficiency (NUE) by enabling controlled nutrient release and enhanced delivery to plants.^12^

Among nanomaterials investigated for fertilizer applications, calcium phosphates (CaPs), particularly hydroxyapatite (HA) and amorphous calcium phosphate (ACP), have attracted significant interest as alternatives to conventional phosphorus fertilizers.^13^ Compared to highly soluble phosphate salts such as triple superphosphates (TSP) or diammonium phosphate (DAP), HA and ACP exhibit lower solubility and pH-responsive dissolution behavior, resulting in a more controlled release and improved NUE.^14,15^

Similarly, urea, the most widely used nitrogen fertilizer, exhibits high solubility and rapid dissolution, which leads to substantial nitrogen losses.^16^ In our previous work, we demonstrated that urea-functionalized ACP nanoparticles effectively regulated nitrogen release and enhanced nitrogen retention in soil.^17^ This nanoenabled formulation significantly slowed urea dissolution compared to free urea, reduced nitrogen leaching, and improved nitrogen uptake in *Zea mays* under grenhouse conditons, while maintaining adequate phosphorus availability. These results highlighted the potential of ACP-based nanomaterials for controlled multinutrient delivery.

Building on this concept, the present study reports a new synthesis route of ACP nanoparticles designed to further explore the environmental and agronomic potential of this material. Specifically, potassium-doped ACP nanoparticles (KACP) were synthesized by replacing sodium-containing precursors commonly used in previous ACP formulations with potassium-based reagents. Unlike sodium, which may persist in the final material and accumulate in the rhizosphere, potentially contributing to soil salinity, potassium is readily taken up by plants and does not pose salinization concerns.^18^ Moreover, potassium is an essential macronutrient and is involved in numerous physiological processes in plants.^19^ This rational substitution transforms the ACP matrix into an intrinsic phosphorus–potassium (PK) nanofertilizer. To achieve a complete NPK formulation, KACP nanoparticles were subsequently functionalized with urea, enabling the controlled release of nitrogen alongside phosphorus and potassium. The resulting material, referred to as KACP-U, was thoroughly characterized and evaluated in greenhouse experiments with *Zea mays*, assessing plant biomass, soil pH, and nutrient leaching relative to conventional fertilizer formulations. Overall, the results of this study demonstrate the rationally designed nanoscale fertilizers represent promising nanomaterial platforms for the development of sustainable fertilizer technologies.

## Materials and Methods

### Materials

All solutions used for synthesis and characterization were prepared using ultrapure water (resistivity >18.2 MΩ·cm at 25 °C) obtained with an Arium Pro purification system (Sartorius, Göttingen, Germany). The following chemicals were used: calcium chloride dihydrate (CaCl_2_·2H_2_O, ≥99.0%), monocalcium phosphate (Ca­(H_2_PO_4_)_2_, >99.0%, hereafter MCP), potassium citrate tribasic monohydrate (K_3_(C_6_H_5_O_7_)·H_2_O, ≥99.0%), potassium phosphate dibasic (K_2_HPO_4_, ≥99.0%), potassium carbonate (K_2_CO_3_, ≥99.0%), nitric acid (HNO_3_, 68.0%), hydrochloric acid (HCl, 37.0%), acetonitrile (CH_3_CN, ≥99.9%), *p*-dimethylaminobenzaldehyde (C_9_H_11_NO, 99.0%, hereafter DMAB), urea (CH_4_N_2_O, ≥99.0%), and potassium chloride (KCl, >99.0%). All chemicals were purchased from Sigma-Aldrich (St. Louis, MO, USA).

### Synthesis of Potassium-Doped Amorphous Calcium Phosphate (KACP)

KACP nanoparticles were synthesized by modifying the method reported by Ghribi et al. (Figure 1)^17^ Two precursor solutions (250 mL each) were prepared: Solution A contained CaCl_2_·2H_2_O (0.82 M) and K_3_(C_6_H_5_O_7_)·H_2_O (0.16 M), while Solution B contained K_2_HPO_4_ (0.48 M) and K_2_CO_3_ (0.80 M). Prior to mixing, Solution B was cooled in an ice bath to prevent premature precipitation. Under continuous vigorous stirring using an overhead stirrer, Solution A was rapidly added to Solution B and mixed for 30 s. The resulting precipitate was collected by centrifugation at 10,000 rpm for 3 min at 4 °C (Sorvall Legend XTR centrifuge, Thermo Fisher Scientific, Waltham, MA, USA), washed three times with ultrapure water, and resuspended in water to a final concentration of 49 mg mL^–1^, determined gravimetrically. An aliquot of the suspension was freeze-dried for physicochemical characterization.

**Figure 1:**
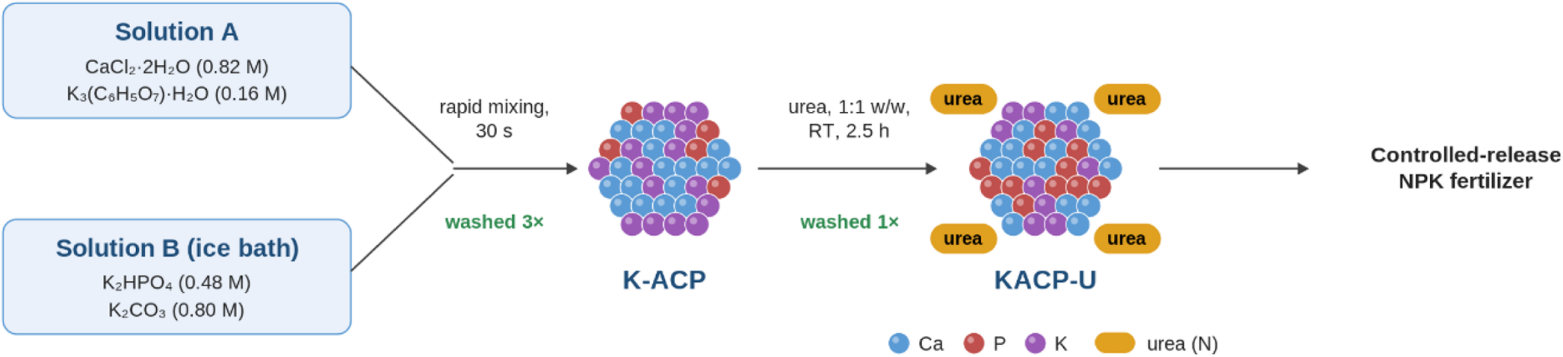
Schematic representation of the synthesis of potassium-doped amorphous calcium phosphate (KACP) from calcium chloride, potassium citrate, potassium phosphate, and potassium carbonate precursors, followed by functionalization with urea to obtain KACP-U. Calcium is shown in blue, phosphate in red, potassium in purple, and urea in orange.

### Functionalization of KACP with Urea (KACP-U)

KACP nanoparticles were functionalized with urea via surface adsorption following a method adapted from Kottegoda et al., who originally developed this approach for hydroxyapatite (HAP) (Figure 1).^20^ Briefly, an aqueous urea solution (49 mg mL^–1^) was added to the KACP suspension in a 1:1 weight ratio. The resulting mixture was stirred at room temperature for 2.5 h. The functionalized material (KACP-U) was recovered by centrifugation under the same conditions described above, washed once with ultrapure water to remove unbound urea, and freeze-dried prior to analysis.

### Materials Characterization

Powder X-ray diffraction (PXRD) patterns were recorded using a D8 Advance diffractometer (Bruker, Karlsruhe, Germany) equipped with a LynxEye position-sensitive detector. Cu Kα radiation (λ = 1.54178 Å) was generated at 40 kV and 40 mA. Data were collected over a 2θ range of 15–60°, with a step size of 0.02° and a counting time of 0.5 s per step.

Fourier-transform infrared (FT-IR) spectra were acquired using a Nicolet iS5 spectrometer equipped with an iD7 attenuated total reflectance (ATR) accessory (Thermo Fisher Scientific, Waltham, MA, USA). Spectra were recorded at a resolution of 2 cm^–1^ by averaging 64 scans over a spectral range of 4000–450 cm^–1^.

Elemental quantification of calcium, phosphorus, and potassium was performed by inductively coupled plasma optical emission spectrometry (ICP-OES) using an Agilent 5100 spectrometer (Agilent Technologies, Santa Clara, CA, USA). For plant tissue analysis, 100 mg of dried material was digested in 4 mL HNO_3_ (68 wt %) at 80 °C for 3 h and subsequently diluted with ultrapure water to a final acid concentration of 2.0 wt % HNO_3_. The same digestion procedure was applied to KACP samples by dissolving 10 mg of material in 2.0 wt % HNO_3_. Liquid samples collected from leaching experiments (4 mL) were acidified with 90 μL of HNO_3_ (68 wt %) to reach a final acid concentration of 2.0 wt %. Calibration curves were prepared using multielement standard solutions, and accuracy and recovery (≥90%) were verified for each analytical run using matrix-matched standards and a certified reference material (NIST SRM 1643f). Nitrogen content in dry materials was determined using a FlashSmart NC Soil Elemental Analyzer (Thermo Fisher Scientific, Waltham, MA, USA) and used to estimate urea content. Approximately 10 mg of each sample was analyzed in triplicate. Urea concentration in leachates was quantified using a DMAB-based colorimetric assay, following the method of Giraldo and Rivas.^21^ Briefly, 1.8 mL of a 20 mmol L^–1^ DMAB solution prepared in acetonitrile was mixed with 1 mL of sample and 37 μL of concentrated HCl (37 wt %). After 10 s of mixing, absorbance was measured at 422 nm using a Lambda 35 UV–Vis spectrophotometer (PerkinElmer, Waltham, MA, USA). Urea concentrations were determined from a calibration curve showing excellent linearity (R^2^ > 0.995) over the working range.

Sample morphology was examined by field-emission Scanning Electron Microscopy (FE-SEM) using a ΣIGMA instrument (ZEISS NTS GmbH, Oberkochen, Germany) coupled to an energy-dispersive X-ray microanalyzer (INCA Energy 450 × 3, Oxford Instruments, Abingdon-on-Thames, UK). FE-SEM micrographs were acquired at an accelerating voltage of 20 keV in InLens mode, while EDS spectra were acquired in secondary electron mode, determining chemical mapping of randomly located particles by accumulation of 5 scan frames. Specific surface area (SSA) was determined by N_2_ adsorption using the Brunauer–Emmett–Teller (BET) method using a Sorpty 1750 instrument (Carlo Erba, Milan, Italy).

### Simulated Leaching Experiment

Soil column leaching experiments were conducted to evaluate nutrient release from KACP-U under accelerated leaching conditions. Two treatments were investigated: (i) KACP-U and (ii) a positive control (CTRL), consisting of a physical mixture of MCP, urea, and KCl. Both treatments supplied identical nutrient inputs corresponding to 333 mg P, 205 mg of N, and 165 mg K per kg of dry soil, corresponding to a balanced nutrient composition equivalent to KACP-U. Experiments were carried out using vertical glass columns with internal dimensions of 40 mm × 200 mm. Each column was packed with 300 g of dry soil previously sieved to <2 mm and gently compacted to ensure uniform packing. The soil used was classified as sandy loam according to the US Department of Agriculture (USDA) classification and exhibited slightly alkaline pH (8.1, measured in a 1:2.5 soil-to-water extract), 2.14% organic matter (Walkley–Black method), total nitrogen of 1.4 g kg^–1^ (Kjeldahl method), and a C/N ratio of 9. Available phosphorus content was 45 mg P_2_O_5_ kg^–1^ (Olsen method), the cation exchange capacity (CEC) was 10.8 mequiv 100 g^–1^ (BaCl_2_ method) and the particle size distribution was 13.5% clay, 32.4% silt, and 54.1% sand (ISSS method). Overall, the soil exhibited intermediate fertility type, with relatively low phosphorus availability, and moderate water-holding capacity, typical of sandy loam textures. A hydrophilic cotton plug was placed at the bottom of each column to prevent soil loss. A continuous flow of ultrapure water (24 mL h^–1^) was supplied throughout the entire duration of the experiment (48 h) using a peristaltic pump (Ismatec, Fisher Scientific, Pittsburgh, PA, USA), corresponding to a simulated rainfall intensity of approximately 458 mm day^–1^. The rainfall equivalence was calculated based on the relation 1 mm rainfall = 1 L m^–2^, following the standard hydrological convention.^22^ Experiments were performed at room temperature (20–25 °C) in triplicate (*n* = 3). Leachates were collected at 1, 2, 3, 4, 5, 6, 24, 25, 26, 27, 28, 29, 30, and 48 h. Urea concentrations were determined using the colorimetric assay described above, while phosphorus, potassium, and calcium concentrations were quantified by ICP-OES.

Release kinetics of urea, calcium, phosphorus, and potassium were analyzed by fitting experimental data to four commonly applied kinetic models: zero-order, first-order, Higuchi, and Korsmeyer-Peppas.^23,24^ Kinetic fitting was performed using the normalized release fraction (*M_t_*/*M_∞_*), and model parameters were obtained by nonlinear least-squares regression using Origin Pro 2023 (OriginLab Corp., Northampton, MA, USA). The applied models are described by the following equations:

Zero-order model

1 $$\frac{M_{t}}{M_{\infty }}=k_{0}t$$

First-order model

2 $$\frac{M_{t}}{M_{\infty }}=1-e^{-k_{1}t}$$

Higuchi model

3 $$\frac{M_{t}}{M_{\infty }}=k_{H}t^{1/2}$$

Korsmeyer-Peppas model

4 $$\frac{M_{t}}{M_{\infty }}=k_{KP}t^{n}$$

where *M*_*t*_ and *M*_*∞*_ represent the cumulative amount of nutrient released at time *t* and at equilibrium, respectively. The parameters *k*_0_, *k*_1_, and *k*_*H*_ are the release constants for the zero-order, first-order model and Higuchi model, respectively, while *k*_*KP*_ and *n* are the kinetic constant and release exponent of the Korsmeyer-Peppas model. Model performance was evaluated based on the coefficient of determination (R^2^) and residual analysis.

### Greenhouse Experiment

A greenhouse experiment was conducted on corn (*Zea mays*, hybrid bicolor Sh2 “BOLT XR F1,” Johnny’s Selected Seeds, Winslow, ME, USA) to evaluate the performance of KACP-U compared with two control treatments: (i) a positive control (CTRL), consisting of soil amended with a physical mixture of MCP, urea, and KCl, and (ii) a negative control (BLK), consisting of untreated soil. Fertilized treatments supplied 333 mg P per kg of dry soil. To ensure nutrient equivalence with KACP-U, urea and KCl were added to MCP in the positive control to provide the same nitrogen and potassium inputs, corresponding to 205 mg N and 165 mg K per kg of dry soil. Based on its phosphorus content (8.8 wt %), KACP-U was applied at approximately 3.8 g per kg of dry soil, equivalent to about 0.38 wt % relative to the soil mass (corresponding to ca. 1.1 g of KACP-U per pot containing 300 g of soil).

Corn seeds were germinated for 5 days in Petri dishes and subsequently transplanted into pots containing a soil mixture composed of 10% Lockwood soil (CAES Lockwood Farm soil, Hamden, CT), 80% Griswold soil (Griswold, CT, USA), and 10% vermiculite (v/v), added to improve porosity and water retention. Fertilizers were suspended in 15 mL of ultrapure water and applied evenly to the soil surface as a slurry 3 weeks after transplanting. The experiment lasted 51 days under greenhouse conditions. At harvest, plants were gently washed with tap water, and roots and shoots were separated and weighed. Dry biomass was determined after oven-drying at 65 °C for 7–10 days until constant mass.

During the experimental period, the greenhouse was maintained under a mixed natural and artificial lighting regime with supplemental lighting. Natural daylight decreased progressively from approximately 12.5 h day^–1^ in early September to ∼10 h day^–1^ in late November (New Haven, CT, 41.3°N). Red-spectrum lamps were activated at sunset each evening for 2 h, yielding a total photoperiod of approximately 14.5 h at the start of the experiment and ∼12 h at its conclusion. Temperature data recorded by the greenhouse monitoring system showed an average daily temperature of approximately 23.5 °C, with maximum values ranging from 28 to 35 °C and minimum values between 13 and 20 °C. Irrigation was performed by top watering: the soil was first brought to saturation, and once percolation began, an additional 50 mL of tap water was applied to ensure leachate generation and collection for analysis. Irrigation events were carried out on days 1, 4, 7, 10, and 20 following fertilizer application, and leachates were collected at each irrigation event to assess nutrient leaching.

To simultaneously assess plant growth, soil pH dynamics, and nutrient leaching behavior within a single integrated experimental design, leachates were collected from both planted and unplanted pots at each irrigation event. Soil pH and electrical conductivity (EC) were monitored at regular intervals using a calibrated pH Tester (HI981030, Hanna Instruments, RI, USA) and a conductivity meter (HI98331, Hanna Instruments, RI, USA) to track changes in soil chemical properties throughout the experiment. Each treatment comprised seven replicate pots containing plants (to provide sufficient statistical power for biomass and elemental analysis) and four replicate pots without plants (to serve as unvegetated controls for leaching and pH measurements), with one plant per pot and 300 g of soil per pot. Pots were 5-in. square plastic nursery pots with an approximate volume of 2 L.

Leaching percentages were calculated according to the following equation:

$$\frac{\mathrm{Total mg leached}-\mathrm{mg leached from BLK}}{\mathrm{mg of the element in the applied material}}\times \mathrm{100}$$ 5

where BLK represents baseline leaching. For treatments with plants, baseline leaching corresponds to pots containing plants without fertilizer addition, while for treatments without plants it corresponds to pots containing neither plants nor added material.

### Statistical Analysis

Pairwise comparisons between KACP and KACP-U were performed using Welch’s *t* test (unequal variances assumed, two-tailed, *p* < 0.05), which was preferred over Student’s *t* test given the heterogeneity of variances between groups. Differences among the three greenhouse treatments (KACP-U, CTRL, and BLK) were assessed using one-way ANOVA followed by Tukey’s HSD post hoc test (*p* < 0.05). All statistical analyses were performed using OriginPro 2023 (OriginLab Corp., Northampton, MA, USA).

## Results and Discussion

### Structural and Chemical Characterization of KACP and KACP-U Materials

In our previous work, CaCl_2_ 2H_2_O (0.10 M), Na_3_C_6_H_5_O_7_ (0.10 M), Na_2_HPO_4_ (0.12 M), and Na_2_CO_3_ (0.20 M) were used as precursors for the synthesis of ACP, yielding a material with a Ca/P molar ratio of 1.64 ± 0.01 and a production yield of 6.60 g L^–1^.^17^ Although this material was successfully functionalized with urea, two major limitations were identified. First, the use of sodium-based precursors raised concerns regarding potential soil salinization, as trace amounts of Na were retained within the amorphous matrix.^17^ Second, the relatively low solubility of sodium salts limited the maximum attainable precursor concentrations, thereby constraining production yield and hindering scalability (Table S1). To overcome these limitations and considering that potassium is an essential macronutrient for plants, the synthesis strategy was redesigned by replacing sodium-based precursors with potassium-based analogues and systematically optimizing precursor molar ratios. This modification allowed the use of substantially higher precursor concentrations due to the high solubility of potassium salts, thereby increasing the nanoparticle production yield per unit volume of solution while simultaneously eliminating the sodium incorporation into the final material. The revised synthesis approach was therefore designed to achieve three concurrent objectives: (i) increasing production yield while reducing precursor losses, (ii) mitigating potential soil salinity risks associated with residual sodium, and (iii) enabling the development of an NPK fertilizer system.

In the optimized formulation, CaCl_2_·2H_2_O (0.82 M) and K_3_(C_6_H_5_O_7_)·H_2_O (0.16 M) were reacted with K_2_HPO_4_ (0.48 M) and K_2_CO_3_ (0.80 M). Compared to the previous system, absolute precursor concentrations were increased several-fold, reflecting the improved solubility of potassium salts (Table S1). The Ca/P molar ratio in the precursor solution increased from 0.83 to 1.70, a value closer to the expected composition of the precipitated ACP phase. In the previous system, despite the low Ca/P ratio in solution, the resulting solid exhibited a Ca/P ratio of 1.64, indicating incomplete phosphorus incorporation and significant phosphorus loss during synthesis. By increasing the precursor Ca/P ratio, phosphorus incorporation was enhanced, yielding a final solid with a Ca/P ratio of approximately 2.0 and markedly reducing phosphorus losses.

Simultaneously, the Ca/citrate molar ratio was increased from 1 to 5. Because citrate strongly complexes Ca^2+^ ions and can inhibit ACP precipitation, its reduced relative concentration diminished this inhibitory effect and promoted more extensive particle formation.^25^ As a result of the combined optimization of precursor chemistry, concentrations, and molar ratios, the production yield increased to 66 g L^–1^, corresponding to a an approximately 10-fold enhancement relative to the previous formulation (Table 1). In addition, potassium was successfully incorporated into the KACP structure at levels of approximately 7 wt %, eliminating sodium-related salinity concerns and intrinsically transforming the material into a phosphorus–potassium (PK) nanofertilizer precursor suitable for subsequent urea functionalization.

**Table 1:** Chemical Composition of KACP and KACP-U

Sample	Ca (wt.%)*	P (wt.%)*	Ca/P	N (wt.%)^§^	K (wt.%)*
KACP	22.8 ± 1.1^a^	8.6 ± 0.5^a^	2.05 ± 0.01^a^	-	7.1 ± 0.2^a^
KACP-U	22.0 ± 0.7^a^	8.8 ± 0.3^a^	1.94 ± 0.01^a^	4.01± 0.01	1.6 ± 0.1^a^

a Data are expressed as the mean ± standard deviation (*n* = 3). Different letters indicate statistically significant variations between samples within the same column (Welch’s *t* test, *p* < 0.001). Nitrogen was not present in KACP and therefore was not subjected to statistical comparison. *Quantified by ICP-OES; ^§^Quantified by NC Soil Elemental Analyzer.

Upon functionalization with urea, the resulting material (KACP-U) exhibited a nitrogen loading of 4.01 ± 0.01 wt %, comparable to that previously obtained for the sodium-based analogue (ACP-Urea, 4.61 ± 0.1 wt %). These values indicate a similar nitrogen-loading capacity for the two materials, suggesting that substitution of sodium with potassium does not significantly affect nitrogen incorporation.

The KACP material exhibited a markedly lower specific surface area (38.0 m^2^ g^–1^) compared to the sodium-based ACP previously reported (200.0 m^2^ g^–1^).^26^ Despite this difference, urea loading was not adversely affected, as evidenced by the comparable nitrogen contents measured for KACP-U and ACP-Urea. These findings indicate that urea incorporation is likely governed by specific interactions with active sites within the amorphous matrix rather than by nonspecific physisorption processes. This further suggests that the availability of specific binding sites per gram of material is comparable in the two systems.

Elemental analysis by ICP-OES, combined with Rietveld refinement of PXRD data, revealed that pristine KACP contained 7.05 ± 0.18 wt % potassium. In contrast, KACP-U exhibited a lower potassium content (1.6 wt %), likely due to partial potassium release during the additional aqueous treatment step required for urea functionalization. This behavior is consistent with partial ion exchange or dissolution processes occurring at the particle surface under aqueous conditions.

The Ca/P molar ratios measured for KACP and KACP-U (Table 1) differed slightly but significantly (2.05 ± 0.01 vs 1.94 ± 0.01, *p* < 0.001). Although statistically significant, the absolute variation remains modest and is unlikely to reflect substantial changes in the bulk phase. Instead, this decrease in Ca/P ratio upon urea functionalization may arise from minor surface rearrangements or partial dissolution-reprecipitation events occurring during the aqueous urea adsorption step.

PXRD analysis (Figure 2a) indicates that both KACP-based samples are predominantly amorphous. In both cases, a broad diffraction halo centered at approximately 30° 2θ is observed, which is characteristic of the amorphous phase.^26^ In the case of pristine KACP, four diffraction peaks are also visible at 28.26°, 40.51°, 50.16°, and 58.46° 2θ. These reflections were attributed to residual KCl that was not completely removed during the washing step.^27^ Rietveld refinement allowed quantification of the KCl phase in KACP, corresponding to 3.21 wt.% KCl (equivalent to 1.68 wt.% K), in good agreement with the residual potassium content measured in the KACP-U material. By contrast, the sodium-based ACP synthesized in our previous study showed complete removal of the corresponding NaCl byproduct. This difference can reasonably be attributed to the substantially higher synthesis yield achieved in the potassium-based route, which results in a more concentrated reaction mixture and reduces the efficiency of the washing process in completely removing soluble salts. For KACP-U, KCl-related diffraction peaks were no longer detected, suggesting that the additional aqueous washing step performed after urea functionalization was effective in removing residual soluble salts. Instead, a weak diffraction peak appeared at 22.2° 2θ, which can be assigned to the (110) plane of crystalline urea present as a discrete crystalline phase rather than adsorbed onto the nanoparticles.^28^

**Figure 2:**
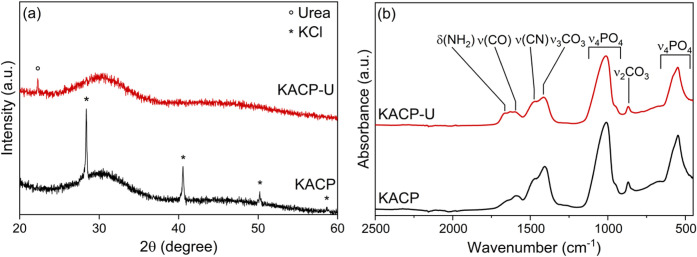
(a) PXRD patterns of KACP-U and KACP and (b) FT-IR spectra of KACP-U and KACP.

The FT-IR spectrum of KACP (Figure 2b) shows broad and poorly resolved absorption bands typical of amorphous calcium phosphate. Bands observed at approximately 560 and 1050 cm^–1^.^29^ These spectral features are consistent with the established characteristics of citrate-stabilized ACP containing carbonate ions.^30^

Compared with the sodium-based ACP previously reported,^17^ a notable difference in the relative intensity of the carbonate bands with respect to the phosphate bands was observed. In the present KACP sample, the carbonate-to-phosphate band intensity ratio was approximately 0.53, whereas a value of 0.21 was reported for the sodium-based material. This difference is plausibly attributed to the reduced washing efficiency associated with the high-concentration synthesis conditions adopted for KACP, which may lead to enhanced retention of carbonate species within or on the surface of the amorphous phase.

The FT-IR spectrum of KACP-U (Figure 2b) shows, in addition to the bands described above associated with the pristine KACP, distinct absorption features characteristic of urea. Specifically, bands observed at approximately 1672 and 1620 cm^–1^ are attributed to the C=O stretching vibration (νC=O) and to coupled asymmetric and symmetric bending modes of amine groups (δasNH_2_ and δsNH_2_) associated with C–N stretching (νCN), respectively. Additional contributions in the 1550–1600 cm^–1^ region may arise from overlapping NH_2_ deformation modes.^31–33^ The presence of these characteristic bands confirms the association of urea at the KACP surface and is consistent with the spectral features reported for urea-functionalized ACP materials in our previous work.^17^ In our previous study, deconvolution analysis of the FT-IR spectra of urea-functionalized ACP revealed shifts of the characteristic urea vibrational bands, particularly those associated with NH_2_ bending modes, suggesting specific interactions between urea amino groups and electrophilic Ca^2+^ sites exposed on the amorphous calcium phosphate surface. Given that KACP retains the amorphous calcium phosphate structure and accessible Ca^2+^ sites, a similar mechanism of urea–Ca^2+^ interaction can be reasonably proposed for KACP-U. Although citrate molecules are retained on the KACP surface and their possible contribution to urea stabilization cannot be completely excluded, no direct evidence is currently available to support a predominant role of citrate–urea interactions.

The morphology of KACP and KACP-U was investigated by field-emission scanning electron microscopy (FE-SEM) (Figure 3). In both cases, the materials consist of agglomerates of spherical nanoparticles with an average size of approximately 75 nm. No significant morphological differences were observed between KACP and KACP-U. FE-SEM images of KACP-U did not reveal the presence of secondary particles or morphologically distinct crystalline domains attributable to urea, indicating that the sample morphology is dominated by the amorphous calcium phosphate nanoparticles. The specific surface area (SSA), determined by the BET method, was comparable for the two materials (38.07 m^2^ g^–1^ for KACP and 36.78 m^2^ g^–1^ for KACP-U), consistent with their similar size. EDS elemental maps (Figure S1) confirmed the composition of the samples and successful incorporation of potassium, showing a homogeneous distribution of Ca, P, K, and O within the nanoparticle matrix. Nitrogen was not detected in KACP-U; however, EDS has intrinsically limited sensitivity toward low atomic number elements, particularly at low concentrations, making the detection of nitrogen amounts below 5 wt.% challenging due to low X-ray emission efficiency and matrix effects.^34^

**Figure 3:**
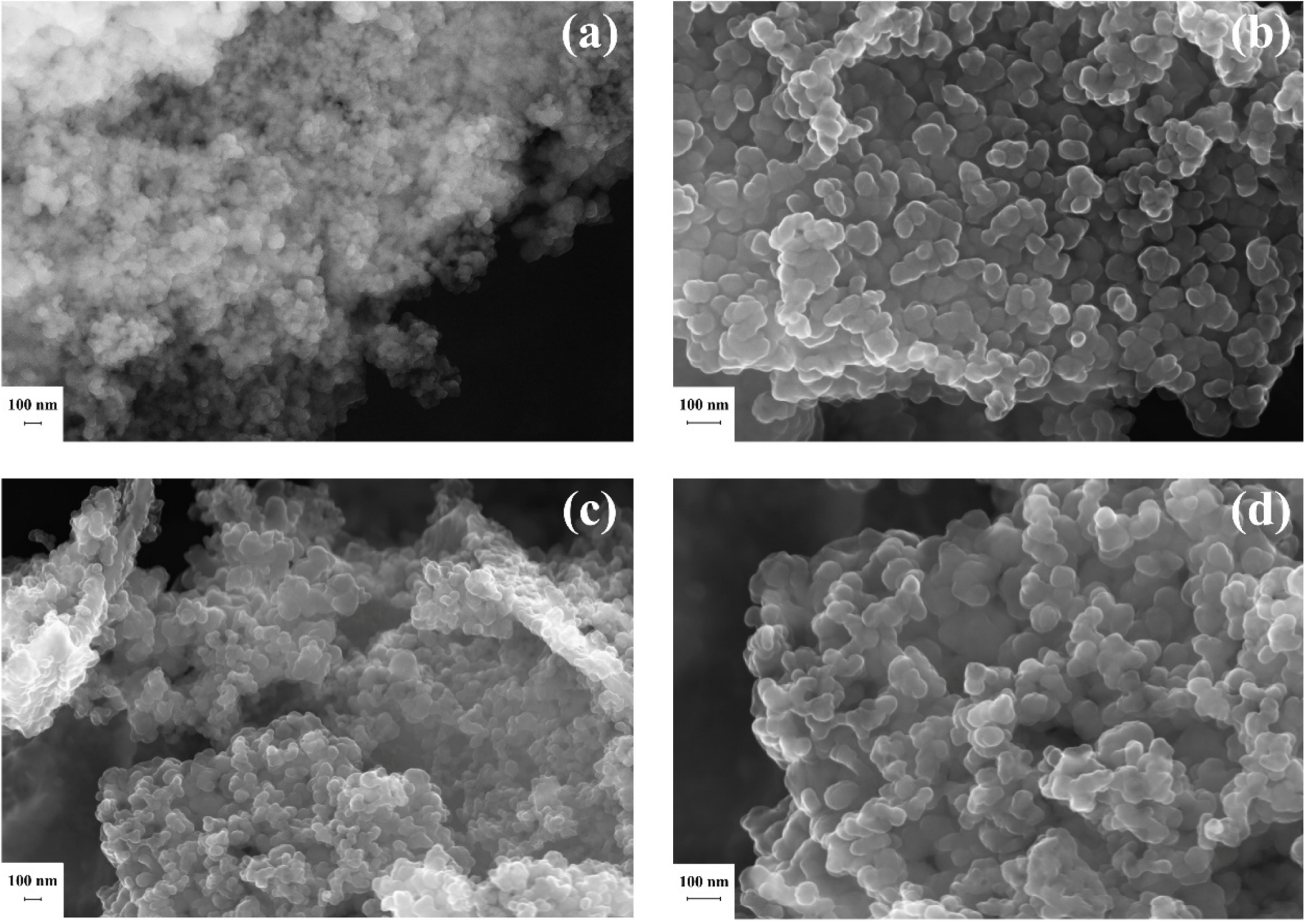
FE-SEM micrographs of (a, b) KACP and (c, d) KACP-U at different magnifications.

### Soil Column Leaching Experiments

Soil column leaching experiments were performed to investigate the release behavior of the major macronutrients under severe simulated rainfall conditions (Figure 4). The leaching flow rate was intentionally set to a high value, corresponding to approximately 458 mm of rainfall per day, to simulate an extreme leaching scenario and accelerate the comparative screening of the developed materials. Such an accelerated approach provides insights into fertilizer behavior under severe water exposure conditions.

**Figure 4:**
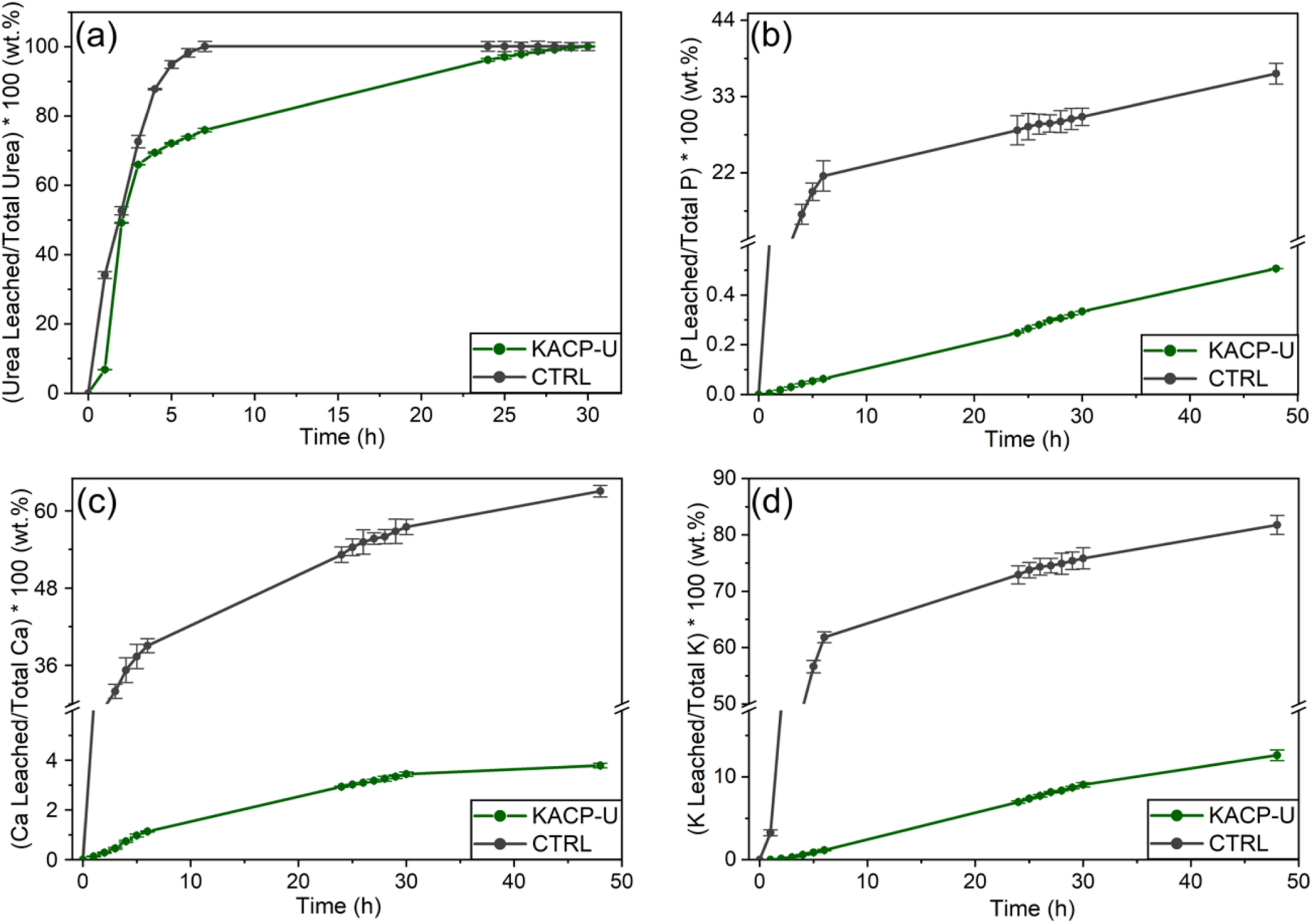
Leaching behavior of KACP-U (green) and the positive control (CTRL, gray), consisting of a physical mixture of potassium chloride, urea, and monocalcium phosphate. Cumulative percentage of nutrient leached over time, expressed as wt.% relative to the total nutrient content applied, for (a) urea, (b) phosphorus, (c) calcium, and (d) potassium. Data are reported as mean ± standard deviation (*n* = 3).

Under these conditions, urea from the control treatment (CTRL) was completely leached within the first 6 h. In contrast, KACP-U exhibited a markedly slower and more gradual urea release, extending over approximately 30 h. Phosphorus release was also strongly reduced for KACP-U, with only about 0.5 wt.% of the applied phosphorus detected in the leachate after 48 h, compared to approximately 35% for the control. Similar trends were observed for calcium and potassium. After 48 h, calcium leaching reached approximately 4% for KACP-U compared to about 64% for the control, while potassium leaching amounted to approximately 10% and 80%, respectively.

The kinetic fitting of the leaching curves is reported in Figure S2, and the corresponding fitting parameters are summarized in Table S2. Kinetic analysis was performed to provide a comparative description of nutrient release behavior between the CTRL and KACP-U. For all investigated nutrients, leaching profiles of the CTRL were consistently well described by a first-order kinetic expression, with coefficients of determination (R^2^) exceeding 0.96. This behavior is consistent with rapid dissolution processes governed primarily by concentration gradients, as expected for highly soluble fertilizer components such as urea, monocalcium phosphate, and potassium chloride.

In contrast, nutrient release from KACP-U occurred more gradually and could be satisfactorily described by different kinetic models, including zero-order, first-order, and Korsmeyer–Peppas expressions, depending on the nutrient. The comparable goodness-of-fit obtained for these models suggests that nutrient release from KACP-U is not dominated by a single kinetic regime, but rather reflects a constrained, matrix-controlled process associated with the amorphous calcium phosphate carrier.

For urea, the first-order kinetic constant obtained for CTRL (*k*_1_ = 0.440 h^–1^) was higher than that observed for KACP-U (*k*_1_ = 0.276 h^–1^), consistent with the faster leaching of free urea compared to urea incorporated onto the ACP matrix. A similar trend was observed for calcium, for which the first-order rate constant was more than four times higher for CTRL (*k*_1_ = 0.230 h^–1^) than for KACP-U (*k*_1_ = 0.050 h^–1^), reflecting the rapid dissolution of calcium from monocalcium phosphate compared to its gradual release from the amorphous matrix.

The most pronounced difference was observed for phosphorus. The first-order rate constant for phosphorus release from the CTRL (*k*_1_ = 0.169 h^–1^) was nearly 2 orders of magnitude higher than that obtained for KACP-U (*k*_1_ = 17.4 × 10^–3^ h^–1^), quantitatively supporting the extremely low phosphorus leaching observed for KACP-U in the column experiments.

Potassium release from KACP-U exhibited a more complex behavior, with different kinetic models providing comparable goodness-of-fit. In this case, fitting results should be interpreted with caution, as the application of multiparameter models to a limited data set may lead to overfitting. Notably, the first-order model still provided an excellent description of potassium release from KACP-U (R^2^ ≈0.99), indicating a release rate approximately 33 times slower than that measured for the CTRL.

Overall, kinetic analysis corroborates the experimental observations and confirms that KACP-U exhibits a strongly constrained and regulated release of all investigated nutrients compared to the conventional fertilizer formulation, resulting in substantially reduced leaching losses under severe rainfall conditions.

### Greenhouse Pot Experiment

#### Plant Growth

In the greenhouse experiment conducted on corn (*Zea mays*), treatment with KACP-U resulted in a significantly higher root dry biomass compared to both the negative control (BLK) and the positive control (CTRL) (Figure 5a). Specifically, root dry weight reached 0.59 ± 0.12 g for KACP-U, compared to 0.17 ± 0.08 g for BLK and 0.40 ± 0.13 g for CTRL (*p* < 0.05). This pronounced enhancement in root biomass indicates improved belowground development under KACP-U treatment.

**Figure 5:**
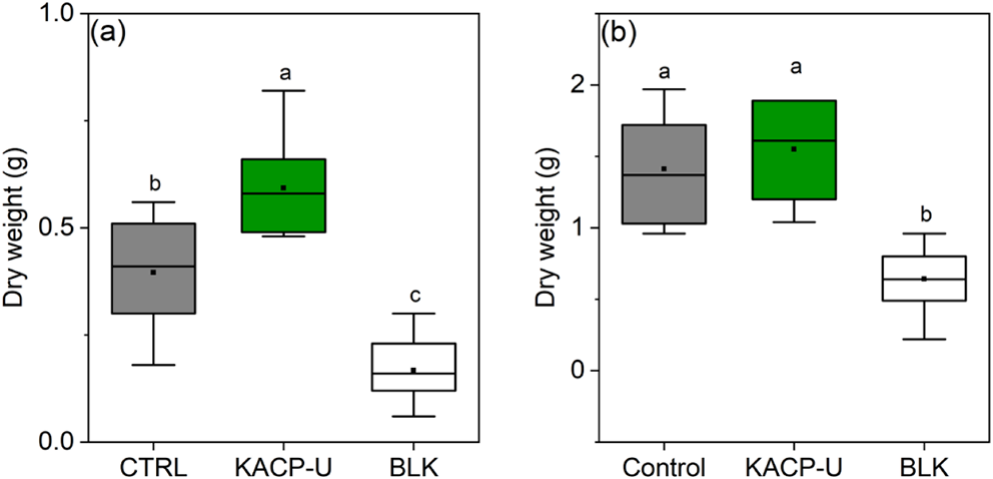
Dry weight of (a) roots and (b) shoots of corn (*Zea mays*) grown under different treatments: KACP-U, positive control (CTRL), and negative control (BLK). Bars represent mean values and error bars indicate standard deviation. Columns labeled with different letters denote statistically significant differences (*p* < 0.05), as determined by one-way ANOVA followed by Tukey’s HSD test.

In contrast, no statistically significant difference in shoot dry biomass was observed between KACP-U and CTRL (Figure 5b). Nevertheless, both fertilized treatments exhibited significantly higher shoot and root dry weights compared to BLK, confirming the positive effect of nutrient supplementation relative to unfertilized soil. The combination of comparable shoot biomass and enhanced root development observed in the KACP-U treatment suggests a shift toward preferential biomass allocation belowground, which may support improved nutrient and water acquisition.

### Elemental Composition of Roots and Shoots

The elemental composition of root and shoot tissues from plants treated with KACP-U, CTRL, and BLK is reported in Figure 6 and reveals treatment-dependent variations in nutrient uptake.

**Figure 6:**
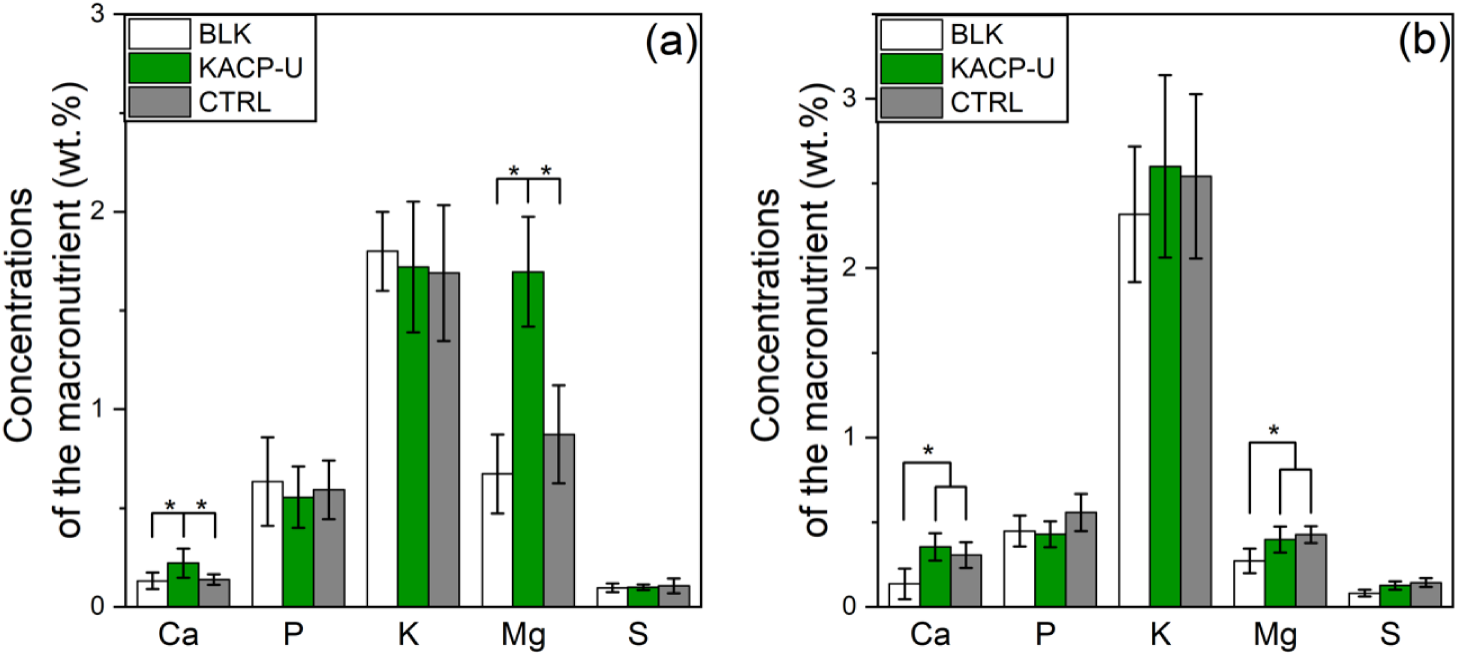
Nutrient concentrations in (a) roots and (b) shoots of corn (*Zea mays*) grown under different treatments: BLK, KACP-U, and CTRL. Elements showing statistically significant differences (*p* < 0.05) are indicated with an asterisk (*).

In root tissues (Figure 6a), plants treated with KACP-U exhibited significantly higher calcium and magnesium concentrations compared to both BLK and CTRL (*p* < 0.05), indicating enhanced delivery and uptake of these nutrients associated with the KACP-U formulation. In contrast, potassium, phosphorus, and sulfur concentrations did not differ significantly among treatments, suggesting comparable uptake of these nutrients across fertilized and unfertilized conditions.

In shoot tissues (Figure 6b), both KACP-U and CTRL treatments resulted in significantly higher calcium concentrations compared to BLK, confirming increased calcium bioavailability under fertilized conditions. Magnesium concentrations in shoots were also significantly higher for KACP-U relative to BLK. No statistically significant differences were observed for phosphorus, potassium, or sulfur among treatments.

These results are consistent with those reported in our previous study, in which urea-functionalized ACP similarly promoted calcium and magnesium accumulation in maize tissues without significantly altering phosphorus uptake.^17^ The reproducibility of these trends further supports the conclusion that KACP-U not only reduces nutrient losses but also facilitates targeted macronutrient delivery, particularly for calcium and magnesium, while maintaining balanced overall nutrient acquisition and shoot biomass.

### Soil pH and Electrical Conductivity Analysis

Measurements of soil pH and electrical conductivity provide insight into the mechanisms underlying the observed increase in root biomass and, more broadly, into the environmental interactions of the KACP-U formulation. As shown in Figure 7a, soil treated with KACP-U exhibited values of electrical conductivity higher than those of BLK and comparable to those measured for CTRL, indicating a similar overall availability of soluble nutrients in the soil solution.

**Figure 7:**
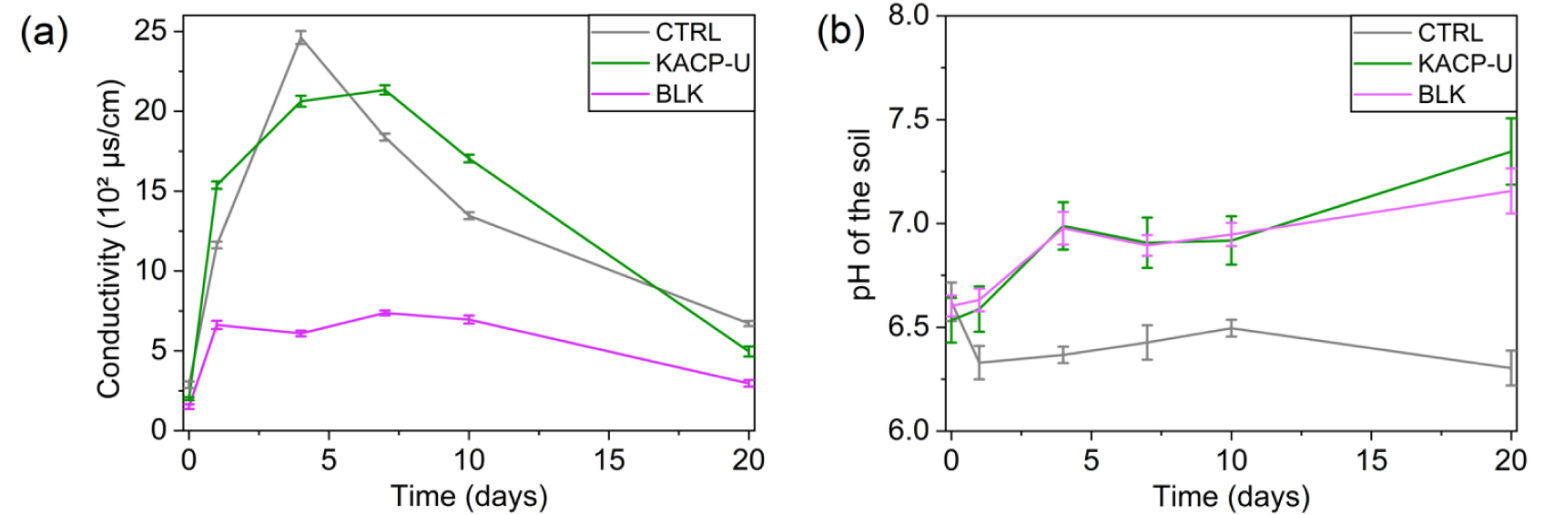
Soil (a) electrical conductivity and (b) pH measured during the greenhouse experiment with corn (*Zea mays*) under different treatments: KACP-U, CTRL, and BLK.

In contrast, notable differences were observed in soil pH (Figure 7b). Soil treated with KACP-U maintained pH values ranging from neutral to slightly acidic (approximately 6.5–7.3), closely matching those measured for BLK. Conversely, CTRL-treated soil showed a consistent shift toward more acidic conditions (approximately 6.2–6.5). These results indicate that KACP-U provides nutrient availability comparable to that of conventional fertilization while mitigating soil acidification, a well-recognized concern associated with the prolonged use of conventional mineral fertilizers.^35^

The observed stabilization of soil pH can be plausibly attributed to the buffering behavior of calcium phosphate-based materials. Under acidic conditions, the dissolution rate of amorphous calcium phosphate increases, leading to the release of orthophosphate species that can consume protons and partially counteract pH decreases.^36^ By moderating soil acidification, KACP-U may contribute to preserving soil chemical stability and to maintain a more favorable rhizosphere environment for root development, consistent with the enhanced root biomass observed in greenhouse experiments.

Additional insight was obtained by comparing soil pH trends in pots with and without plants (Figure S3). Between days 4 and 10 of the experiment, the presence of corn plants was generally associated with a modest increase in soil pH across all treatments, suggesting a plant-mediated influence on soil chemical conditions. Such effects may arise from rhizosphere processes including root exudation, nutrient uptake, and ion exchange.^37^

This observation is particularly relevant in the context of engineered fertilizer systems based on calcium phosphates, as nutrient release from ACP phases is strongly pH-dependent. Improved understanding of plant-mediated pH variations over time may therefore enable more precise tuning of fertilizer formulations, allowing nutrient release profiles to be better aligned with soil conditions and plant demand while minimizing adverse environmental impacts such as soil acidification.

### Leaching Experiment in Pots with and without Plants

Building on the results obtained from the soil column experiments, which demonstrated the strong ability of KACP-U to mitigate nutrient losses under severe leaching conditions, leachates were collected from the greenhouse pot experiment described above to assess nutrient release dynamics under greenhouse conditions. Both KACP-U and CTRL were evaluated in the presence and absence of plants (Figure 8). Importantly, no additional experiment was performed: the leachates analyzed here were collected from the same pots used for plant growth during each irrigation event.

**Figure 8:**
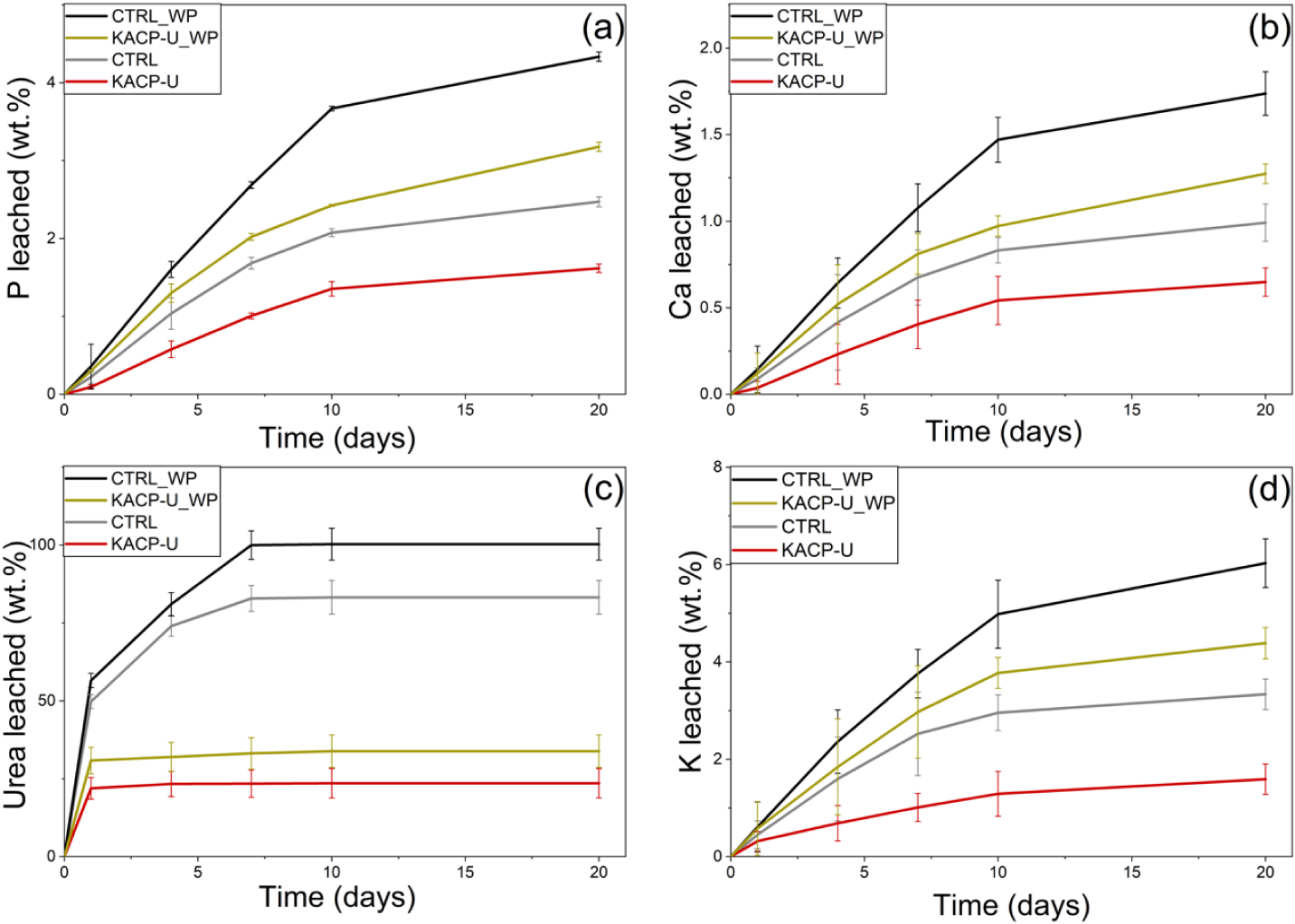
Percentage of leached (a) urea, (b) phosphorus, (c) calcium, and (d) potassium from different treatments: CTRL without plants (CTRL_WP), KACP-U without plants (KACP_WP), CTRL with plants (CTRL), and KACP-U with plants (KACP-U). Leaching percentages were calculated according to eq 5.

With respect to urea leaching (Figure 8a), the CTRL treatment without plants exhibited nearly complete urea loss within the first week, confirming the high solubility and mobility of free urea in soil.^16^ When plants were present, urea leaching in the CTRL treatment decreased to approximately 80% of the applied nitrogen, most likely as a result of partial nitrogen uptake by the root system and/or incorporation within soil microbial biomass.

In contrast, KACP-U markedly limited urea losses under both conditions. In pots containing plants, cumulative urea leaching remained stable at approximately 20–25 wt.% of the applied nitrogen throughout the experimental period, corresponding to an approximate 75% urea reduction compared to the CTRL. Considering that maize nitrogen demand is relatively low during early vegetative growth and increases progressively at later stages, the substantial urea retention observed for KACP-U is primarily attributed to the physical confinement of urea within the ACP matrix and to interactions with soil constituents, rather than to immediate plant uptake. This sustained urea availability, together with the limited nitrogen requirements of maize prior to silking, likely explains the comparable biomass production observed between KACP-U and the positive control despite the substantially reduced urea leaching.^38^

Urea in soil undergoes dynamic transformation processes, including nitrification, microbial immobilization, and other soil nitrogen dynamics which represent relevant mechanistic pathways that may help further explain these observations and warrant further investigation. A similar behavior was observed for phosphorus, calcium, and potassium (Figure 8b–d). Over the 20-day experimental period, KACP-U consistently exhibited lower cumulative leaching than the CTRL, irrespective of the presence of plants. For potassium, CTRL released 6.02 ± 0.50 wt.% in the absence of plants and 3.33 ± 0.31 wt.% in planted pots, whereas KACP-U released only 3.17 ± 0.06 wt.% and 1.59 ± 0.31 wt.%, respectively. Comparable trends were observed for phosphorus and calcium. In pots containing plants, KACP-U released 1.61 ± 0.05 wt.% of phosphorus and 0.64 ± 0.08 wt.% of calcium, while in the absence of plants these values increased to 3.18 ± 0.06 wt.% and 1.27 ± 0.05 wt.%, respectively. By comparison, the CTRL treatment leached 2.47 ± 0.06 wt.% of phosphorus and 0.99 ± 0.10 wt.% of calcium in planted pots, and 4.33 ± 0.06 wt.% of phosphorus and 1.74 ± 0.12 wt.% of calcium in pots without plants.

Overall, nutrient losses from the CTRL treatment were approximately 27–35% higher than those observed for KACP-U under comparable conditions. The presence of plants reduced nutrient leaching for both treatments, although the magnitude of this effect varied depending on the nutrient type. For KACP-U, plant presence reduced phosphorus and calcium losses by approximately 50%, while reductions of around 43% were observed for the CTRL. Potassium leaching decreased by approximately 50% in the presence of plants for both materials.

These results indicate that while plant uptake contributes to limiting nutrient losses, the consistently lower leaching values observed for KACP-U under all conditions demonstrate that the material provides an additional nutrient retention mechanism beyond biological uptake alone. In comparison with the soil column experiment, the pot study confirms the same overall behavior under milder hydrological conditions. Although absolute leaching values were lower in pots, likely due to intermittent irrigation and ongoing nutrient uptake by plants, the relative differences between KACP-U and CTRL remained consistent across experimental designs. This consistency demonstrates that the controlled-release properties of KACP-U are preserved under both extreme and under regular irrigation conditions, highlighting its potential to mitigate nutrient dispersion into percolating water while preserving plant growth and development.

## Conclusions

Urea-functionalized amorphous calcium phosphate synthesized using potassium-based precursors represents a rational nanoenabled strategy for the development of sustainable fertilizer formulations. Replacing sodium with potassium represented a step forward toward scalability as it enabled a 10-fold increase in synthesis yield per volume, while mitigating soil salinization risks and transforming the material into a complete NPK nanofertilizer.

Greenhouse and pot experiments provided evidence that KACP-U enhances root biomass, promotes calcium and magnesium accumulation in corn tissues, and markedly reduces nutrient losses compared to conventional fertilization practices. Under the tested greenhouse conditions, phosphorus and calcium leaching were reduced by approximately 35%, while urea losses decreased by about 75%, without inducing soil acidification. These effects are attributed to the pH-responsive behavior of the amorphous calcium phosphate matrix, which enables controlled nutrient release and improved nutrient retention in soil. Further investigation of soil nitrogen transformation processes may provide additional mechanistic insight into fertilizer behavior and support continued optimization of formulation performance.

Overall, KACP-U combines high production efficiency with improved plant growth responses (higher root biomass and root nutrient uptake) together with reduced urea leaching under the tested conditions, supporting its potential as a controlled-release nanostructured NPK fertilizer platform for sustainable agricultural applications. Although the economic viability and industrial scalability of KACP-U production were beyond the scope of the present study, the synthesis yield achieved here (66 g L^–1^) represents a 10-fold improvement over previously reported ACP formulations and constitutes an important step toward large-scale production. Future studies will focus on comprehensive techno-economic evaluation, large-scale manufacturing feasibility, and field-scale validation under realistic agronomic conditions. In addition, long-term studies across different soil types and climatic regimes, together with detailed characterization of bioavailable phosphorus fractions and nitrogen transformations, will support further optimization of the formulation and assessment of its agronomic and environmental performance.

## Supplementary Material



## Notes

Solubility of sodium- and potassium-based precursor salts (Table S1); EDS elemental maps of KACP and KACP-U (Figure S1); kinetic fitting of the soil-column leaching data and corresponding fitting parameters (Figure S2 and Table S2); soil pH in planted and unplanted pots (Figure S3) (PDF)
