# Supplementary material for: Potassium-Doped Amorphous Calcium Phosphate Nanoparticles Functionalized with Urea as a Sustainable Controlled-Release NPK Fertilizer
Source: ACS Appl Nano Mater. 2026 Jul 23;9(31):14926–37. doi: 10.1021/acsanm.6c02420 (PMC13471757; doi:10.1021/acsanm.6c02420)
Supplement: Supplementary file 1 [file an6c02420_si_001.pdf]

Supporting Information

Potassium-Doped Amorphous Calcium Phosphate Nanoparticles Functionalized with Urea as a Sustainable Controlled-Release NPK Fertilizer

Salem Ghribi[1,2], Lorenzo Degli Esposti[3], Blaire Steven[4], Nubia Zuverza-Mena[4], Jing Yuan[4], Jason C. White[4], Deb P. Jaisi[5], Alessio Adamiano[6], Michele Iafisco[6,*]

[1]Institute of Science, Technology and Sustainability for Ceramics (ISSMC), National Research Council (CNR), 48018 Faenza (RA), Italy

[2]Department of Mathematical, Physical and Computer Sciences, University of Parma, 43124 Parma, Italy

[3]Dipartimento di Chimica e Chimica Industriale, Università degli Studi di Genova, 16146 Genova, Italy

[4]Connecticut Agricultural Experiment Station, New Haven, CT 06511, USA

[5]Interdisciplinary Science and Engineering Laboratory, Department of Plant and Soil Sciences, University of Delaware, Newark, DE 19716, USA

[6]Institute of Nanostructured Materials (ISMN), National Research Council (CNR), 40129 Bologna, Italy

*Correspondence to: michele.iafisco@cnr.it, Tel: +39 0516399133.

Table S1. Solubility of sodium- and potassium-based precursor salts at 25°C

Precursor type	Compound	Solubility (g/L, 25°C)
Sodium	$Na_3(C_6H_5O_7)\cdot 2H_2O$	420
	$Na_2HPO_4\cdot 2H_2O$	150
	Na_2CO_3	215
Potassium	$K_3(C_6H_5O_7)\cdot 1H_2O$	1600
	K_2HPO_4	1670
	K_2CO_3	1120

Note: Values illustrate the substantially higher solubility of potassium-based precursors relative to their sodium counterparts, enabling the use of higher precursor concentrations during synthesis.

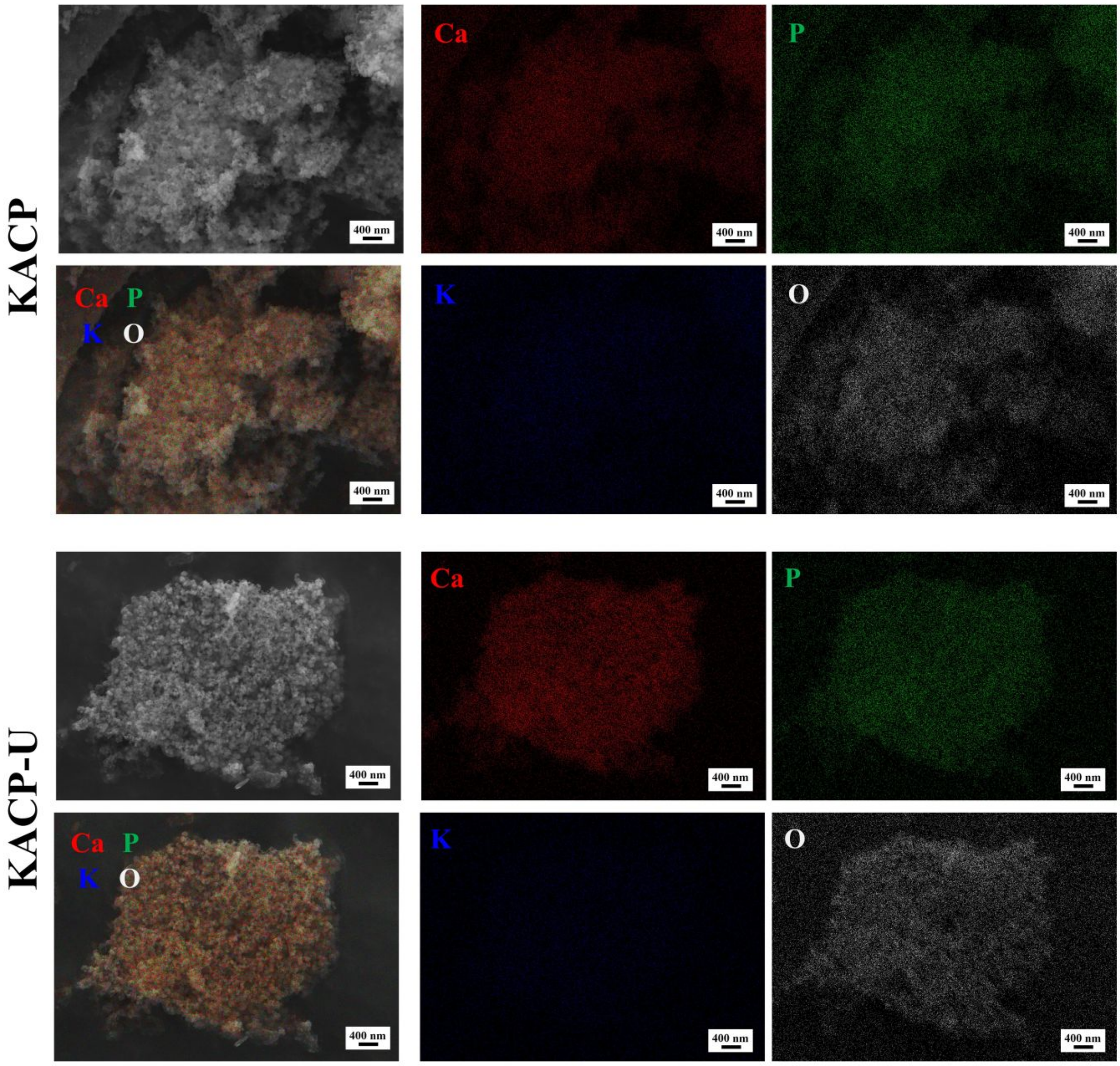

Figure S1. EDS elemental maps of KACP and KACP-U showing the distribution of Ca, P, K, and O, together with the overlay of the elemental maps on the corresponding FE-SEM micrographs.

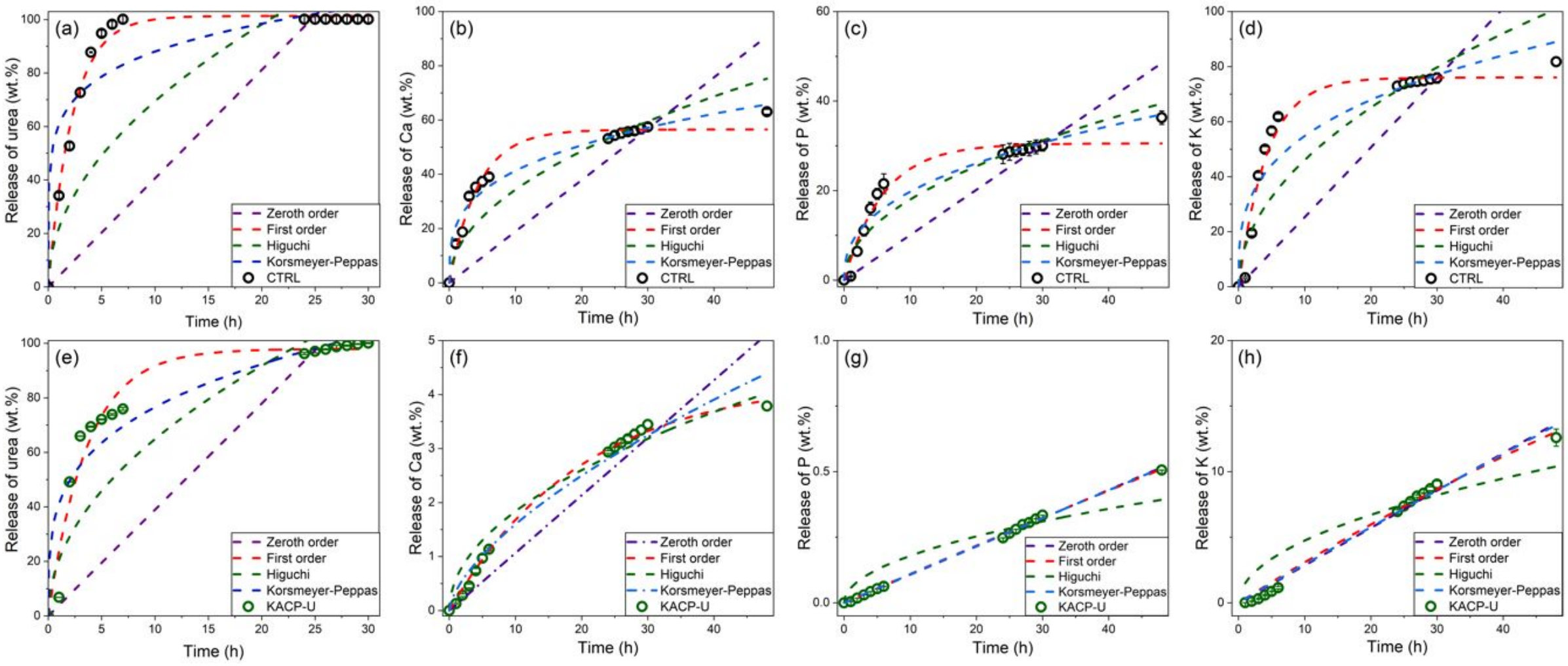

Figure S2. Kinetic fitting of the soil column leaching experiments for the positive control (CTRL): (a) urea, (b) calcium, (c) phosphorus, and (d) potassium; and for KACP-U: (e) urea, (f) calcium, (g) phosphorus, and (h) potassium.

Table S2. Kinetic fitting parameters for the release of urea, calcium, phosphorus, and potassium from the positive control (CTRL) and KACP-U obtained from soil column leaching experiments.

Model	Parameter	KACP-U	CTRL
**Urea Release**			
Zero-order	R^2	-0.067	-1.218
	k_0 (h^{-1})	3.90 ± 0.46	4.06 ± 0.63
First-order	R^2	0.961	0.99
	k_1 (h^{-1})	0.276 ± 0.026	0.440 ± 0.022
Higuchi	R^2	0.694	0.152
	k_H ($h^{-1/2}$)	20.5 ± 1.2	22.0 ± 1.9
Korsmeyer–Peppas	R^2	0.894	0.940
	k_{KP} (h^{-n})	0.411 ± 0.05	0.411 ± 0.05
	n	0.271 ± 0.041	0.108 ± 0.030
**Calcium Release**			
Zero-order	R^2	0.898	0.131
	k_0 (h^{-1})	0.107 ± 0.005	1.89 ± 0.20
First-order	R^2	0.997	0.980

	k_1 (h^{-1})	0.050 ± 0.003	0.230 ± 0.016
Higuchi	R^2	0.951	0.827
	k_H ($h^{-1/2}$)	0.581 ± 0.020	10.9 ± 0.5
Korsmeyer–Peppas	R^2	0.974	0.970
	k_{KP} (h^{-n})	(3.61 ± 0.64) × 10^{-3}	0.209 ± 0.015
	n	0.645 ± 0.052	0.2961 ± 0.0225
**Phosphorus Release**			
Zero-order	R^2	0.998	0.546
	k_0 (h^{-1})	0.0108 ± 0.0001	1.01 ± 0.09
First-order	R^2	0.999	0.961
	k_1 (h^{-1})	(17.4 ± 1.35) × 10^{-3}	0.169 ± 0.020
Higuchi	R^2	0.883	0.914
	k_H ($h^{-1/2}$)	0.0568 ± 0.0034	5.70 ± 0.21
Korsmeyer–Peppas	R^2	0.998	0.936
	k_{KP} (h^{-n})	(1.12 ± 0.08) × 10^{-4}	0.0787 ± 0.0118
	n	0.400 ± 0.045	0.400 ± 0.046
**Potassium Release**			
Zero-order	R^2	0.985	0.159
	k_0 (h^{-1})	0.286 ± 0.006	2.53 ± 0.29
First-order	R^2	0.988	0.968
	k_1 (h^{-1})	(7.01 ± 4.28) × 10^{-3}	0.234 ± 0.023
Higuchi	R^2	0.853	0.769
	k_H ($h^{-1/2}$)	1.50 ± 0.10	14.6 ± 0.8
Korsmeyer–Peppas	R^2	0.986	0.881
	k_{KP} (h^{-n})	(3.12 ± 0.68) × 10^{-3}	0.269 ± 0.043
	n	0.975 ± 0.062	0.309 ± 0.050

Notes:

- Kinetic fits were performed using the normalized release fraction M_t/M_∞ which is dimensionless.
- Rate constants are reported with units consistent with the applied kinetic expressions: h^{-1} for zero-order and first-order models, $h^{-1/2}$ for the Higuchi model, and h^{-n} for the Korsmeyer–Peppas model.
- The release exponent *n* is dimensionless.
- Negative R^2 values indicate poor model applicability and are reported for completeness.
- All values are reported as mean ± standard deviation (n = 3).

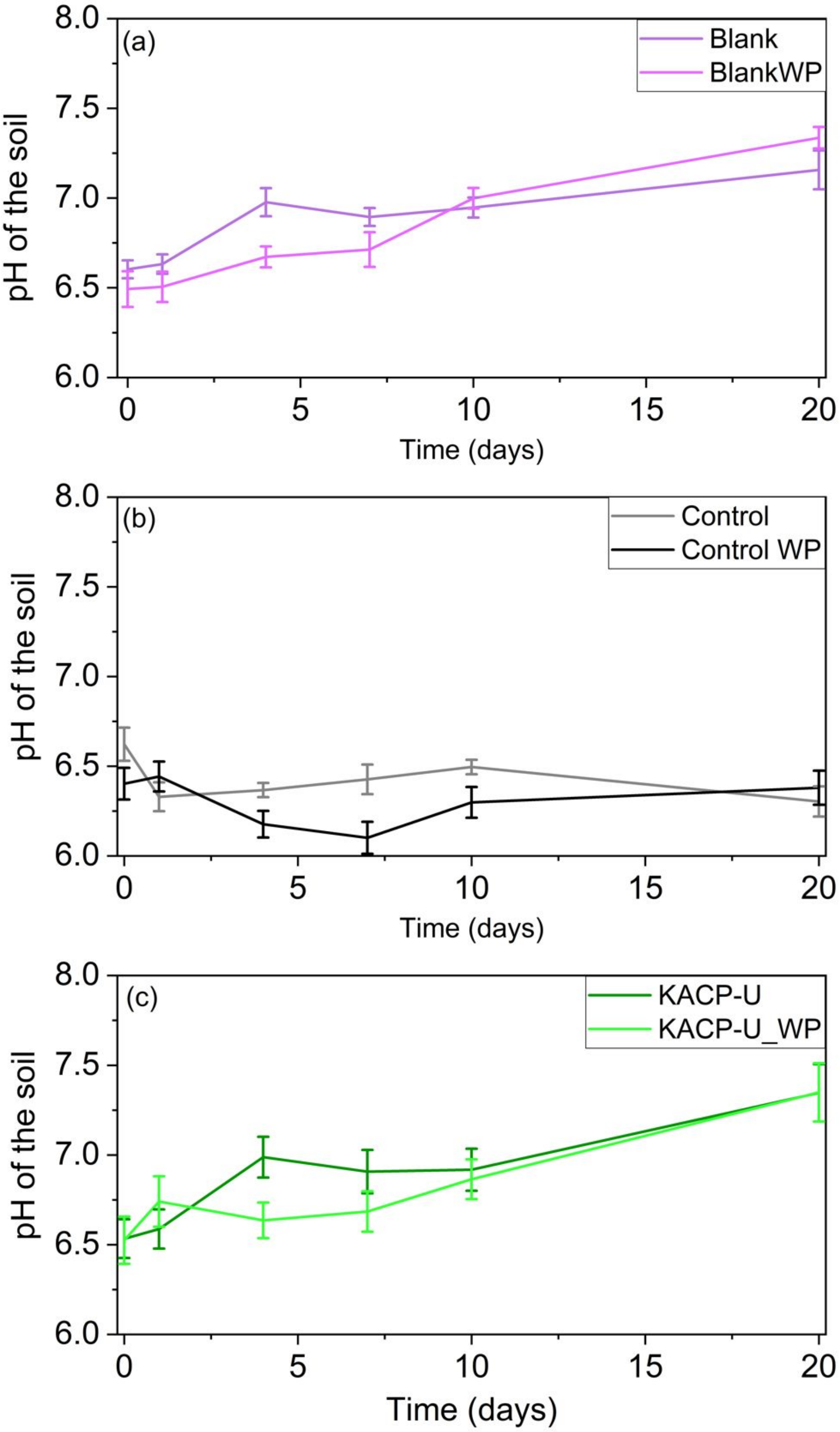

Figure S3. Soil pH measured during the greenhouse experiments for (a) BLK and BLK_WP, (b) CTRL and CTRL_WP and (c) KACP-U and KACP-U_WP.
